# A Two-Stage Classification Method for Improved Fault Detection in Wind Turbines Based on SCADA Data

**DOI:** 10.3390/s26123865

**Published:** 2026-06-17

**Authors:** Jiazhi Dai, Mario Rotea, Nasser Kehtarnavaz

**Affiliations:** 1 Department of Electrical and Computer Engineering, University of Texas at Dallas, Richardson, TX 75080, USA; jiazhi.dai@utdallas.edu; 2 Center for Wind Energy, University of Texas at Dallas, Richardson, TX 75080, USA; rotea@utdallas.edu; 3 Department of Mechanical Engineering, University of Texas at Dallas, Richardson, TX 75080, USA

**Keywords:** wind turbine fault detection, two-stage unsupervised and supervised fault detection, fault detection based on SCADA data

## Abstract

Fault detection is essential for the reliable operation of wind turbines. Traditional supervised methods for fault detection based on SCADA data face highly imbalanced datasets of normal and fault samples. This paper presents a two-stage detection method to address this limitation by integrating unsupervised anomaly detection or classification with supervised classification. In the first stage, the unsupervised classifier of OCSVM, together with two complementary anomaly scores, is used to flag deviations from normal operation or separate abnormal data samples from normal data samples. In the second stage, the supervised classifier of CNN is applied to the detected abnormal data samples to identify fault samples among only these samples, thus enhancing the discrimination capability between normal and abnormal conditions. Experiments on real-world SCADA data show that the introduced two-stage detection method noticeably improves fault detection compared to supervised methods, both in terms of accuracy and missed fault rates.

## 1. Introduction

Wind power constitutes a major source of renewable energy, contributing approximately 7.8% to electricity generation globally [1]. Reliable operation of wind turbines is of critical importance. Turbine faults can lead to downtime, increased maintenance costs, and loss of energy production. Accurate fault detection enables reliable wind turbine operation.

Supervisory Control and Data Acquisition (SCADA) sensors are widely deployed in wind turbines, providing continuous measurements of turbine conditions such as wind speed, power output, temperatures, vibration, and electrical signals [2]. Data from SCADA sensors, called SCADA data, allow addressing fault detection by machine learning in a data-driven manner. Existing machine learning approaches to fault detection involve supervised learning methods. As mentioned in [3,4], these methods require large amounts of labeled data consisting of both normal and fault conditions. In practice, fault-labeled datasets are often scarce, incomplete, or inconsistent. Furthermore, wind turbine data are often highly imbalanced, with normal-operation data samples dominating, making it difficult for supervised models to effectively distinguish fault from normal samples.

To improve fault detection beyond the existing supervised methods, this paper introduces a two-stage classification method by combining unsupervised classification with supervised classification. This two-stage method first identifies SCADA data samples that deviate from normal behavior using only normal data (a single class classification), and then applies a supervised model to those samples that are flagged as not-normal or abnormal samples to identify fault samples among them. By filtering out a large amount of normal data in the first stage, the introduced method reduces the search space for the supervised model to achieve a more accurate fault detection compared to just using supervised classification.

The main contributions in this paper are as follows: (i) Two complementary anomaly scores are introduced for carrying out the unsupervised classification: a feature-based anomaly score capturing deviations in individual SCADA data features, and a correlation-based anomaly score represented as a heatmap characterizing changes in the relationships among SCADA features. For each anomaly score, a one-class learning model is employed to detect abnormal samples. (ii) A two-stage fault detection framework is developed by integrating unsupervised anomaly detection with supervised fault detection to address the severe class imbalance commonly observed in SCADA datasets.

The remainder of the paper is organized as follows. Section 2 reviews related work on wind turbine fault detection. Section 3 presents the introduced methodology of two-stage classification. Section 4 describes the dataset and experimentations performed. The results obtained and their discussion are stated in Section 5, followed by the conclusion in Section 6.

## 2. Related Works

**Figure 1:**
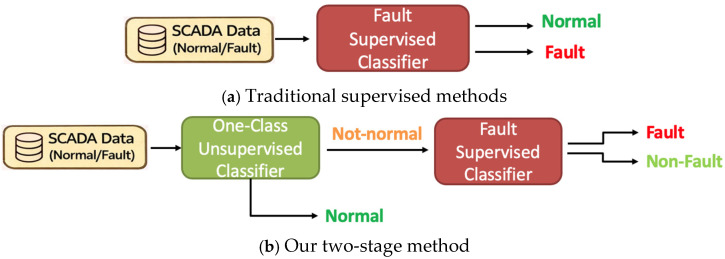
Difference between (**a**) traditional supervised detection methods and (**b**) the developed two-stage detection method. The label “Non-Fault” refers to not-normal samples identified by the first-stage unsupervised classifier that get classified by the second-stage classifier not to be fault samples.

Wind turbine fault detection based on SCADA data has been extensively studied in the literature due to its importance in the reliable operation of wind turbines. Existing machine learning approaches are primarily supervised methods, while in this work, unsupervised learning is used as the frontend to supervised methods. The difference between our two-stage detection method and supervised methods is illustrated in Figure 1.

Early studies on wind turbine condition monitoring mainly relied on statistical approaches. These methods include trend analysis, threshold-based alarms, clustering, and the Normal Behavior Model (NBM) [5]. Trending and threshold-based methods monitor deviations of key features or variables (e.g., temperature, power output) from their expected ranges. While simple and interpretable, as pointed out in [6], these methods often fail to capture complex relationships among SCADA features or variables, making them more susceptible to false alarms and performance degradation. NBM is one of the most widely used methods in monitoring SCADA data. In this approach, a model is trained using normal data to predict expected behavior, and deviations between predicted and observed values get flagged as anomalies. Several variations of NBM have been considered, including regression models, neural networks, and statistical models. These methods offer the advantage of using SCADA data that are readily available and do not require the installation of specialized sensors on a turbine.

The above traditional methods are often limited by their reliance on handcrafted features, which may not generalize well to different fault types. With advancements in machine learning, supervised models have been designed by treating wind turbine fault detection as a classification problem based on labeled data. Conventional machine learning models such as Support Vector Machine (SVM), Random Forest, Xgboost, and Ensemble methods, e.g., [7,8,9], have been used to classify different fault types based on SCADA features. More recently, deep learning models, including Convolutional Neural Network (CNN), Recurrent Neural Network (RNN), and Long Short-Term Memory (LSTM), have been utilized for fault detection, e.g., [10,11,12]. These models learn complex patterns in data and have been shown to provide better performance compared to earlier methods.

The bottleneck in deep learning models is the need for large datasets of labeled normal and fault samples. In practice, as noted in [13], such datasets are scarce and highly imbalanced. Noting that fault events occur infrequently compared to normal operation, it becomes challenging to train supervised models. To address this shortcoming of supervised models, one-class unsupervised methods, which use only readily available normal data, can be considered to lower the search space of supervised models. In [14,15,16,17,18], unsupervised classifiers, including One-Class SVM (OCSVM), AutoEncoder (AE), and LSTM-AE, have been utilized. Despite their advantage of not requiring labeled data, unsupervised methods in general generate more false positives. Essentially, supervised methods are simple but struggle when normal data dominates a dataset, while unsupervised methods ease this shortcoming but are subject to less precision.

In this work, we combine unsupervised and supervised classification to take advantage of the strengths of each of these classifications in order to reach an improved detection outcome as compared to commonly used supervised methods.

## 3. Two-Stage Methodology

**Figure 2:**
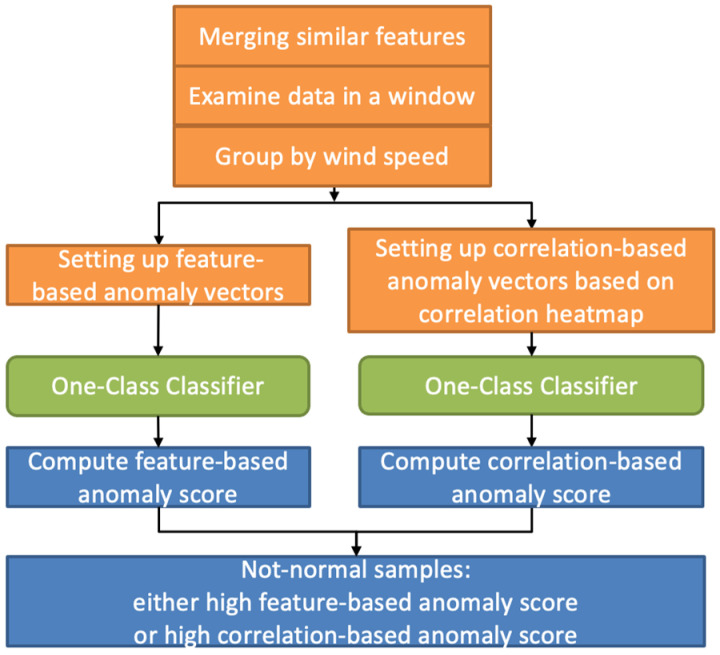
Flowchart of the developed two-stage fault detection method.

The two-stage method presented in this paper aims to automatically detect deviations from the normal state of a wind turbine using the entire SCADA data or measurements. The deviation from normal operation is carried out based on only normal data of a wind turbine that are readily available. As shown in Figure 2, the unsupervised classification is made up of three main steps: (1) data preprocessing, (2) one-class detection, and (3) anomaly score computation. The input consists of multidimensional SCADA data samples collected over time, and the output consists of SCADA data samples identified as deviations from the normal operation state.

Two complementary anomaly scores are computed to quantify deviations from normal behavior. Finally, two one-class learning models are trained on normal data to identify abnormal samples. These samples can be directly used for fault detection or further processed with a supervised classifier to identify fault type.

### 3.1. Data Preprocessing

#### 3.1.1. Feature Merging

A raw SCADA dataset contains many measurements that are physically related and often provide overlapping information. Examples include three-phase electrical variables, multiple temperatures associated with the same component, and vibration measurements collected from closely located sensors. Using all the features increases dimensionality, which in turn makes the anomaly detection process more sensitive to noise. Therefore, a feature merging step is first performed to bring down the dimensionality.

Feature merging is achieved here by the aggregation of very similar physical features. In other words, SCADA features that exhibit both physical consistency and similarity are merged. Physical consistency means that the features correspond to the same component, such as temperatures of different generator phases. Similarity means that these features exhibit similarity over time under normal operating conditions.

**Figure 3:**
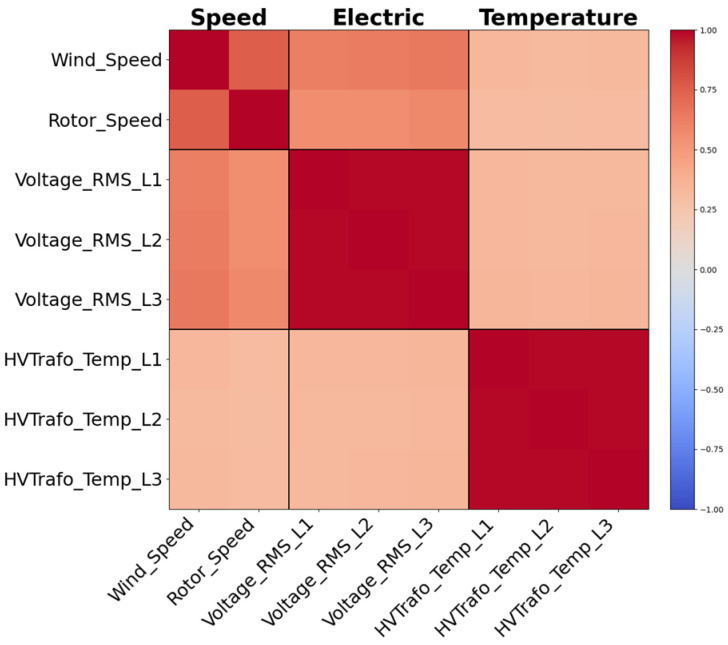
Example illustrating the use of Pearson correlation heatmap to conduct feature merging.

To decide which features to merge, the correlation among them is examined by computing the Pearson correlation coefficient. For a pair of SCADA features $x_{i}$ $and\;x_{j}$, the Pearson correlation coefficient is computed as follows:

(1) $$p_{ij}=\frac{cov\left(x_{i},x_{j}\right)}{\sigma_{i}\sigma_{j}}$$

where $cov\left(x_{i},x_{j}\right)$ denotes the covariance between the two features, $\sigma_{i}$ and $\sigma_{j}$ their standard deviations. As mentioned in [19], features are considered highly correlated when their correlation coefficients are close to 1 under normal operating conditions. In such cases, the features are interpreted as carrying the same information and are therefore suitable for merging. Figure 3 illustrates an example of using the Pearson correlation heatmap to group the SCADA features into speed, electric, and temperature groups. When the features within the same group exhibit strong correlations, they are merged. For example, a subset of features within the temperature group, such as the three transformer temperature measurements, shows strong dependencies or carries the same information, and thus they are merged into a single feature. Similar merging is performed for other feature groups or families. In other words, related features are merged when the following conditions are satisfied: (i) they represent the same physical quantity or the same component from different phases, and (ii) they exhibit very high correlation during normal operation.

Merged features are considered to be the average of the original features. Averaging is adopted here because it preserves the common trend in the same feature group while reducing random fluctuations from individual sensors. In situations where a different merging strategy is more physically meaningful (for example, for vibration signals), root-mean-square values are used.

#### 3.1.2. Window Size Selection

**Figure 4:**
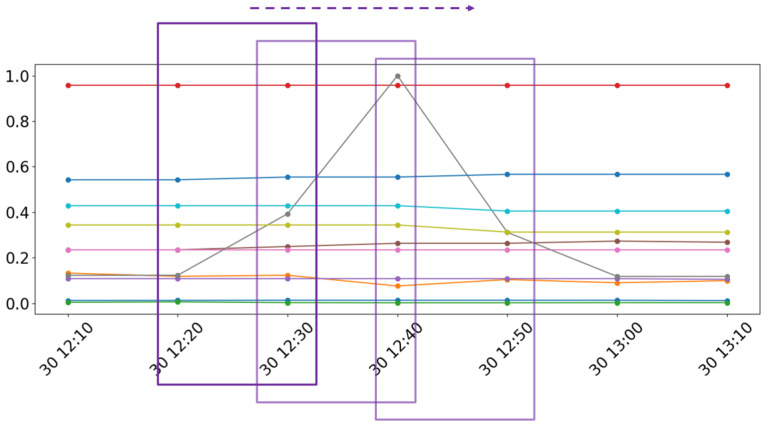
Illustration of sliding window.

To capture temporal changes, the data are examined in a sliding window manner as illustrated in Figure 4. Given a time-series signal *x*(*t*), each sample is constructed as a sequence of measurements over a fixed window of length *T*:

(2) $$X_{t}=\left[x\left(t\right),x\left(t+1\right),\ldots ,x\left(t+T-1\right)\right]$$

where *T* represents the window size. Many abnormal conditions in wind turbines do not appear as a single outlier at one time instant, but rather as a gradual deviation or a persistent abnormal pattern over a time period. If the window is too short, the resulting sample may only reflect instantaneous sensor noise, making it difficult to distinguish abnormal behavior from normal behavior. If the window is too long, abnormal behavior may get diluted by a large amount of normal data within the same window. Therefore, the window size should strike a balance between temporal continuity and preserving anomalous contrast. The selection of window size can be carried out by going from too short to too long windows and then seeing for what window size range more or less the same high-performance outcome is obtained.

#### 3.1.3. Grouping by Wind Speed

**Figure 5:**
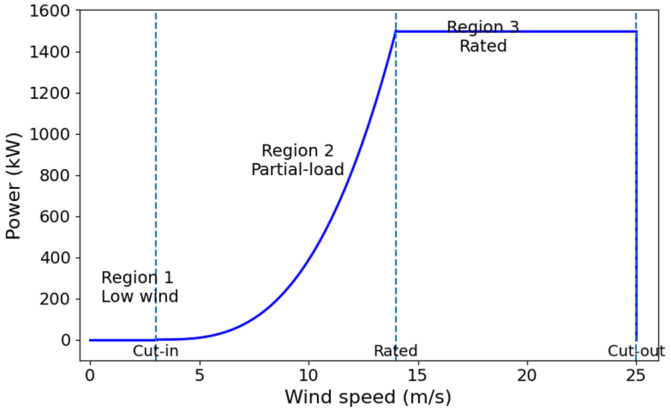
A typical wind turbine power curve.

Under different wind speeds, the relationship between SCADA data features, such as power output, temperature, and vibration, changes considerably. Figure 5 displays a typical power curve of a wind turbine, indicating its power output vs. wind speed.

To address this issue, the dataset is divided into three groups corresponding to the three regions of the power curve. At low wind speeds, typically below or slightly above the cut-in threshold, a turbine generates little or no power. In this region, SCADA data features such as power output, generator temperature, and rotational speed remain relatively low. In the mid wind speed region, the turbine power follows a cubic curve. At high wind speeds, the turbine reaches its rated power output, and this power is regulated to remain constant. In this region, the turbine control system adjusts blade pitch and other components to limit the mechanical load, ensuring a safe operation. Grouping by wind speed helps reduce false alarms caused by variations in turbine operation under different wind speeds. In the subsequent modules of the unsupervised classifier, each wind speed data group is treated independently.

### 3.2. One-Class Detection or Classification Using OCSVM

To detect deviations from normal turbine operation, a one-class classifier is utilized. This classifier is trained exclusively based on only normal data, allowing it to learn the underlying distribution of normal turbine data. Here, OCSVM is used as the one-class classifier as it has the ability to identify outliers without requiring labeled anomaly or abnormal samples.

A sliding window representation $X_{t}\in R^{m\times T}$ is first constructed, where *m* is the number of features and *T* is the window size. For each window, a set of anomaly scores is computed to characterize different types of deviations. These scores are aggregated into a feature vector $z_{t}=\varphi \left(X_{t}\right)$, serving as the input to the OCSVM. Here, $z_{t}$ refers to either ${z_{t}}^{F}$ or ${z_{t}}^{C}$ depending on whether the feature-based or correlation-based OCSVM is used. Transformation for different anomalies is explained in Section 3.3. Given a set of normal samples $\left\{z_{1},z_{2},\ldots z_{n}\right\}$, the OCSVM learns a decision boundary that encloses the great majority of these samples by solving the following optimization problem

(3) $$\underset{w,\xi ,\rho }{\min}\frac{1}{2}{\left\|w\right\|}^{2}+\frac{1}{\nu N}\sum_{t=1}^{N}\xi_{t}-\rho$$

subject to the constraint

(4) $$\left(w\cdot \phi \left(z_{t}\right)\right)\geq \rho -\xi_{t},\xi_{t}\geq 0$$

where the decision function is defined as:

(5) $$f\left(z_{t}\right)=sgn(\left(w\cdot \phi \left(z_{t}\right)\right)-\rho )$$

with $\phi \left(\cdot \right)$ being an RBF (Radial Basis Function) kernel; the parameter ν∈(0,1] specifies the maximum allowable fraction of outliers; N denotes the number of samples, w the normal vector of the decision boundary, and ρ its location in the feature space. The slack variable $\xi_{t}$ is added to allow for minor deviations due to noise. A data sample gets classified as follows:

$$f\left(z_{t}\right)\;\geq \;0\to \;Normal;f\left(z_{t}\right)\;<0\to \;Abnormal$$

In this work, the OCSVM is trained using only normal SCADA data within each wind speed group. The parameter ν is set to 0.05 based on the assumption that abnormal operating conditions constitute only a small fraction of SCADA data. An RBF kernel is employed where its scale parameter is automatically computed from the variance of training data. During inference, each SCADA window is evaluated using the learned decision function, and data samples that fall outside the normal region are identified as not-normal or abnormal samples.

### 3.3. Anomaly Score Computation

The outcome of the one-class classification largely depends on how the SCADA window $X_{t}$ is represented. Different representations reflect different aspects of turbine behavior, and thus different anomalies. Here, two complementary anomaly representations or scores are considered: (i) feature-based anomaly, which captures deviations in individual features, (ii) correlation-based anomaly, which captures changes in relationships among the features. These two representations are used to compute anomaly vectors, which are then fed to the OCSVM model to obtain corresponding anomaly scores.

#### 3.3.1. Feature-Based Anomaly Score

The feature-based anomaly score measures how much individual SCADA features deviate from their normal behavior. For each feature i, its mean $\mu_{i}$ and standard deviation $\sigma_{i}$ are estimated from normal data, i.e., for a given window $X_{i,t}$, the deviation is computed as follows:

(6) $${z_{i,t}}^{F}=\varphi \left(X_{i,t}\right)=\frac{1}{T}\sum_{\tau =1}^{T}\frac{\left|x_{i}\left(t+\tau \right)-\mu_{i}\right|}{\sigma_{i}}$$

The vector ${z_{t}}^{F}=\;\{{z_{1,t}}^{F},\;{z_{2,t}}^{F},...{{,z}_{m,t}}^{F}\}$ indicates the average normalized deviation of all the features over the window. A higher ${z_{i,t}}^{F}$ value indicates that the feature signals deviate more from their normal levels. This vector is then used as the input to the feature-based OCSVM to obtain the feature-based anomaly score. Since anomaly scores are concentrated within a narrow range under normal conditions, an exponential transformation is applied to amplify abnormal behavior. Meanwhile, very small scores are regarded as noise and set to zero.

#### 3.3.2. Correlation-Based Anomaly Score

While a feature-based anomaly score captures individual feature deviations, it does not account for the relationships among features. However, under normal operation, SCADA features exhibit stable correlations. To capture changes in these relationships, a correlation-based anomaly score is considered. First, a baseline correlation matrix $R_{i,j}^{\left(0\right)}\in R_{m\times m}$ is computed using normal data in each wind speed region. Specifically, the data are divided into sliding windows, and for each window k, a correlation matrix is computed as follows:

(7) $$R_{i,j}^{\left(k\right)}=p_{ij}=\frac{cov\left(x_{i},x_{j}\right)}{\sigma_{i}\sigma_{j}}$$

The baseline correlation matrix is then obtained by averaging over *N* windows in the same wind speed region:

(8) $$R_{i,j}^{\left(0\right)}=\frac{1}{N}\sum_{k=1}^{N}R_{i,j}^{\left(k\right)}$$

For each sliding window $X_{t}$, a correlation matrix $R^{\left(t\right)}$ is computed in the same manner. The difference between the current correlation matrix and the baseline correlation matrix is computed. The upper-triangular elements of the difference matrix are extracted and arranged into the following correlation-based anomaly vector:

(9) $${z_{t}}^{C}=\varphi^{C}(X_{t})={vec}_{u}(R^{\left(t\right)}-R^{\left(0\right)})\;$$

where ${vec}_{u}(\cdot )$ denotes the upper-triangular entries. This vector is then used as the input to the correlation-based OCSVM to obtain the correlation-based anomaly score. This score captures relational anomalies; that is to say, even when individual features remain within their normal ranges, abnormal behavior can still appear via changes in their relationships.

#### 3.3.3. Combination or Union of Two Anomaly Scores

After inputting the feature-based and correlation-based anomaly vectors separately into two one-class classifiers, the two corresponding anomaly scores are computed to capture complementary aspects of abnormal turbine behavior. The feature-based anomaly score focuses on individual feature deviations, identifying situations where one or more SCADA data features exceed their normal operating levels. This type of anomaly is typically associated with observable changes in magnitude, such as a sudden increase in bearing temperature or an abnormal drop in power output. In contrast, the correlation-based anomaly score focuses on capturing changes in the relationships among the features. Under normal operation, SCADA features exhibit stable correlations due to the inherent coupling of turbine components. When these relationships are altered, it may indicate abnormal operating conditions even if individual feature values remain within their normal limits. It is to be emphasized that the two anomaly scores are different. Feature-based anomaly scores reflect local deviations, while correlation-based anomaly scores reflect system-level changes. As a result, relying on only one type of anomaly score may not capture all possible abnormalities.

**Figure 6:**
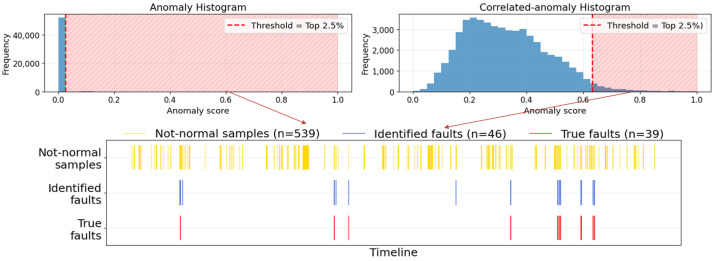
Histograms of anomaly scores; union of feature-based anomaly score and correlation-based anomaly score represented as yellow bars (abnormal samples), blue bars (identified fault samples), and red bars (true fault samples) across the time axis.

In practice, abnormal events are relatively rare and typically account for only a small fraction of SCADA data. For example, as reported in [20], the percentage of anomalous samples is less than 5% of the entire SCADA dataset. A percentile-based selection strategy is thus adopted here. For each anomaly score, a top percentage of samples with the highest scores are selected as abnormal data samples. The final set of abnormal data samples is obtained by taking the union of these two sets. This process is illustrated in Figure 6. In this figure, yellow bars represent abnormal SCADA samples across the time axis, and red bars represent fault samples across the time axis. By using both of the anomaly scores, ideally, abnormal samples should capture all the fault samples; i.e., all the fault samples should be marked as abnormal, and none of the fault samples should be marked as normal.

It is worth pointing out that the unsupervised classifier in Stage 1 is not intended to serve as the final fault detector. Instead, its primary role is to identify samples deviating from normal-operation samples and reduce the severe class imbalance commonly encountered in SCADA datasets. These samples are then used to train and evaluate the Stage-2 supervised classifier. In other words, the unsupervised classifier can be viewed as an anomaly-screening component for conducting supervised classification. Also, the unsupervised classification is applied independently to training and testing, and only the identified abnormal samples are passed to the supervised classifier.

## 4. Experimental Studies

### 4.1. Dataset

Our experimental studies are reported in this section by using the public domain Zenodo Wind Farm B dataset provided in [21]. This dataset contains SCADA data or signals collected from three wind turbines (#27, #34, #77) that exhibit faults. The data is sampled at a 10-min interval and includes a variety of features such as wind speed, power output, and multiple sensor measurements. The dataset consists of normal and fault conditions. It was randomly divided into 70% training, 10% validation, and 20% testing subsets. The validation subset was used for anomaly-threshold sensitivity analysis and window-size selection, whereas the testing subset was reserved exclusively for performance evaluation. Since fault occurrences were highly imbalanced and unevenly distributed throughout the dataset, a fixed-ratio random split was adopted to ensure sufficient fault samples in each subset. To avoid temporal leakage, the training, validation, and testing windows were generated separately, and overlapping windows were used within each subset, but no windows were shared across different subsets. The number of training/validation/testing samples is provided in Table 1.

For the unsupervised model, only normal data was used during training to learn the distribution of normal data. Fault data was exclusively used for evaluation. For the supervised model, both normal and fault data were used for training and testing under the same data split regime. The preprocessing steps, including normalization and feature merging, as described in Section 3, are stated next.

### 4.2. Preprocessing

#### 4.2.1. Input Features

The SCADA features in this dataset reflect the electrical, thermal, and mechanical conditions of the wind turbines. They include voltage and current measurements, generator and transformer temperatures, bearing temperatures, vibration signals, and gearbox-related temperatures, as summarized in Table 1. Since many SCADA variables are collected from multiple phases or closely related components (e.g., L1, L2, L3 phases), strong correlations are observed among these signals. Feature merging was thus performed by aggregating highly correlated features into representative features. Features such as wind speed and wind direction were not merged, while variables like grid frequency, which remain constant over time, were excluded to reduce the dimensionality. All the merged features of the dataset are listed in Table 2.

#### 4.2.2. Candidate Threshold Analysis

To determine an anomaly-selection threshold for the first stage, the degree of class imbalance commonly reported in SCADA datasets was considered. A previous study in [20] reported fault-to-normal ratios of approximately 1:360, 1:490, and 1:2930, corresponding to fault proportions below 0.5% of the total data. A similar fault proportion (approximately 0.5%) was thus considered in our study.

Noting that the objective of the first-stage unsupervised detector is not to directly identify fault samples but rather to identify suspicious samples for further examination by the second-stage supervised classifier, the number of retained candidate samples needs to be higher than the true faults. A sensitivity analysis was conducted using the validation subset. The 2-anomaly-score-based OCSVM was employed, and the retained percentage of not-normal samples for each anomaly score was varied from 0.5% to 5%. To avoid introducing assumptions about the final window size during the threshold selection, a temporary 12-h window was considered. The effect of window size was investigated in the subsequent experiment. For each threshold, both fault coverage and the resulting class imbalance after Stage 1 were examined. The results obtained are shown in Table 3.

The results in Table 3 indicate that increasing the retained percentage improved fault coverage. Although a 5% threshold still achieved the highest fault coverage, it also retained a substantially larger number of normal samples, resulting in a post-filtering normal-to-fault ratio of approximately 19:1. In contrast, a target candidate ratio of approximately 2.5% achieved a substantially lower imbalance ratio while maintaining high fault coverage. Therefore, a target candidate ratio of 2.5% was selected.

#### 4.2.3. Window Size Selection

**Figure 7:**
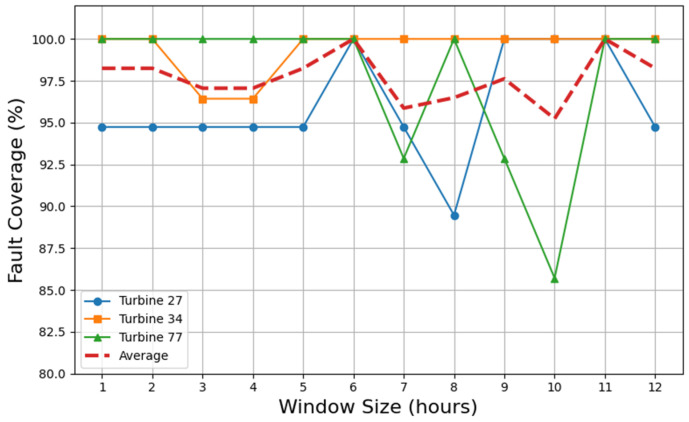
Fault coverage rate for each window size across different turbines.

As discussed in [22], for the SCADA data at a sampling rate of 10 min, the window length needs to be several hours to distinguish between normal and fault samples. Figure 7 presents the fault coverage rates obtained using window lengths ranging from 1 to 12 h.

High fault coverage was achieved across a broad range of window sizes, indicating that the developed framework is relatively robust to the window-length selection. Among the evaluated configurations, both the 6-h and 11-h windows achieved 100 fault coverage. The 6-h window size was selected since it was the shortest window length achieving complete fault coverage, was less susceptible to the influence of missing data and required lower computational cost than longer windows. This window length corresponds to 36 SCADA samples. The slight reduction in coverage for some longer windows is likely due to the dilution of fault-related patterns by a larger proportion of normal operating data within the window.

#### 4.2.4. Grouping by Wind Speed

**Figure 8:**
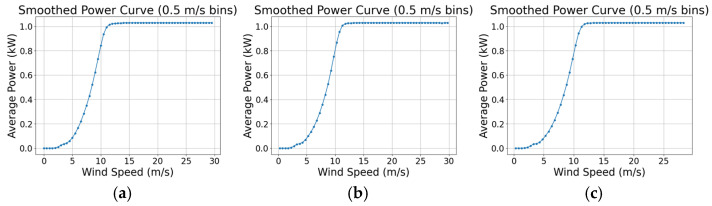
Power curve of the three turbines in the dataset: (**a**) turbine #27, (**b**) turbine #34, (**c**) turbine #77.

To account for different wind speed conditions, the data were grouped based on wind speed. Specifically, the entire dataset was divided into three wind speed intervals or regions (low, medium, and high wind speed regions). The power curves of the three turbines in the dataset are shown in Figure 8. It can be seen that the curves are similar, having the three wind speed regions of (0–2.5 m/s), (2.5–11.5 m/s), and (>11.5 m/s).

#### 4.2.5. Selection of Unsupervised and Supervised Classifiers

For unsupervised classification, several models were evaluated for their ability to distinguish between normal and abnormal conditions. Among the unsupervised models examined (see Table 4), the One-Class Support Vector Machine (OCSVM) was selected because it provided greater discrimination between normal and fault samples. Similar to unsupervised models, NBM only uses normal data for analysis. For a typical NBM, a Linear Regression model in [23] was considered here as a baseline. This model was trained using only normal operating data to obtain the turbine power output, and the absolute prediction residual was used as the anomaly score. The top-ranked residual samples were then selected as abnormal candidates for fault coverage evaluation. For the neural-network models (Autoencoder, LSTM-AE, CNN, and the unsupervised graph representation learning model from [24]), the number of training epochs was fixed at 50, and the learning rate was set to 0.001 for consistency. OCSVM was configured with a radial basis function (RBF) kernel (ν = 0.05) and did not require training epochs or a learning rate. All the models were tested with their best window size. These comparisons show that, except for OCSVM, the neural-network-based unsupervised models did not achieve better fault coverage of the dataset under examination here. This outcome suggests that increasing model complexity does not necessarily improve the Stage-1 fault screening performance.

For supervised classification, several models were evaluated for their ability to distinguish filtered data between non-fault and fault conditions. As shown in Table 5, the Convolutional Neural Network (CNN) provided the fewest missed faults, and it was thus selected for the subsequent experimentations. All models were tested using their respective optimal window sizes. Recent approaches such as KNN and ConvLSTM-Transformer [25,26], which have shown promising performance in related applications, were also evaluated. However, they did not achieve superior performance. XGBoost missed fewer positive samples comparable to those of CNN, but CNN consistently missed fewer fault samples across the three turbines. These results suggest that CNN provided the best balance between fault detection capability and overall classification performance for the SCADA dataset examined.

**Figure 9:**
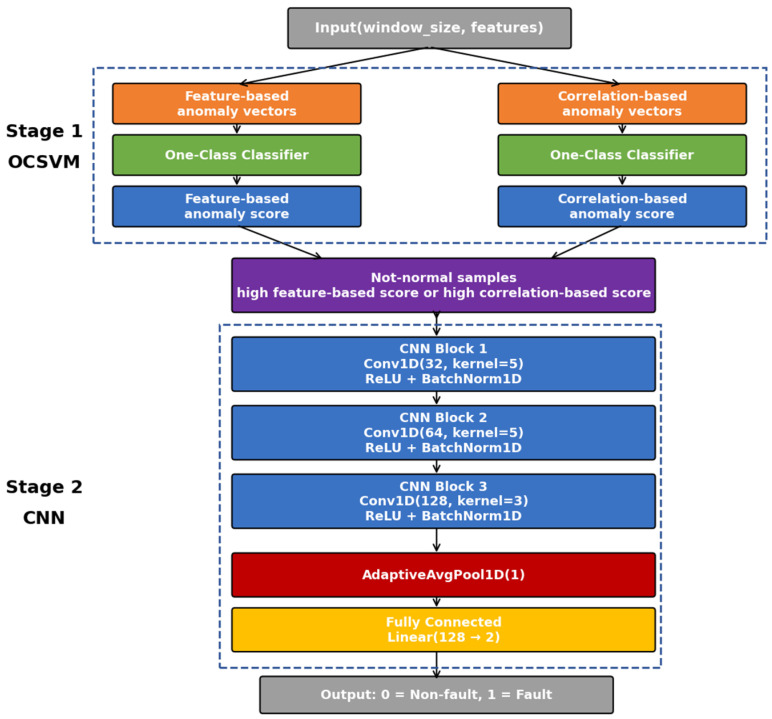
Architecture of the OCSVM+CNN model.

Our two-stage detection thus consists of OCSVM as the unsupervised classifier in stage 1 and CNN as the supervised classifier in stage 2. This combination leverages the strength of unsupervised learning in identifying candidate anomalies and supervised learning in refining fault detection. Figure 9 shows the architecture of the OCSVM+CNN model.

### 4.3. Performance Metrics

#### 4.3.1. Metric for Unsupervised Learning

For the unsupervised classification stage, performance is evaluated using the fault coverage rate, defined as the percentage overlap between detected abnormal samples and true fault samples.

(10) $$\;Fault\;coverage\;rate=\frac{overlap\;between\;abnormal\;and\;fault\;samples}{total\;fault\;samples}$$

This metric measures the ability of the unsupervised anomaly detector or classifier in capturing fault events, regardless of fault type. A higher coverage rate indicates better detection of fault-including anomalies.

#### 4.3.2. Metrics for Supervised Learning

For the supervised classification stage, the following metrics are considered:

(11) $$Missed\;Normal\;Rate\;(MNR)=\frac{Missed\;normal\;samples}{Total\;normal\;samples}$$

This metric measures the proportion of normal samples incorrectly classified as faults (false positives).

(12) $$Missed\;Fault\;Rate\;(MFR)=\frac{Missed\;fault\;samples}{Total\;fault\;samples}$$

This metric evaluates the proportion of fault samples that are not detected (false negatives), which is a critical measure for reliable wind turbine operation. In addition, the commonly used accuracy measure is considered. Accuracy is defined as

(13) $$Accuracy=\frac{TP+TN}{TP+FP+TN+FN}$$

where *TP*, *FP*, *TN*, and *FN* denote true positives, false positives, true negatives, and false negatives, respectively.

## 5. Results and Discussion

### 5.1. Combined Anomaly Score vs. Feature-Based Anomaly Score

This study compared the effectiveness of the feature-based anomaly score versus the combined anomaly score. Table 6 shows the outcome on the test data when using the combined anomaly scores introduced in this paper and the feature-based anomaly scores used in [27,28]. As shown in this table, the combined anomaly scores consistently achieved a fault coverage rate of 100%.

**Figure 10:**
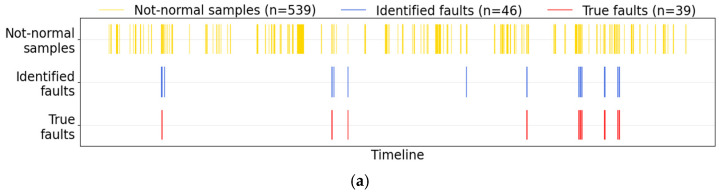

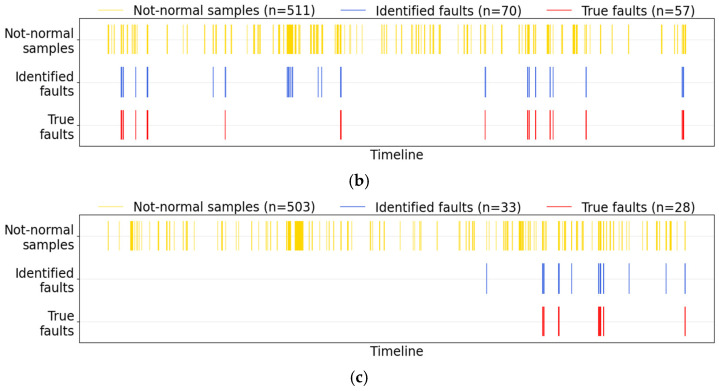
Temporal visualization of detected anomaly samples and fault samples for the three turbines of the dataset: (**a**) #27, (**b**) #34, and (**c**) #77.

As shown in Figure 10, the yellow lines represent abnormal samples detected by the unsupervised classifier, the blue lines represent the faults identified by the supervised classifier, and the red lines indicate the fault samples. The fault samples completely overlap with the detected anomaly samples for all three turbines, indicating that the unsupervised classifier captured all the fault-related deviations. After processing these candidate samples, the CNN in Stage 2 identifies fault events that closely align with the ground-truth faults.

These results demonstrate that the combined anomaly score provides a more comprehensive representation of abnormal behavior and improves the robustness of fault detection. In addition, it indicates that the developed two-stage framework effectively suppresses false alarms while preserving fault-related information, enabling accurate fault detection with a significantly reduced search space.

### 5.2. Two-Stage Method vs. Supervised Method

This study evaluates the main contribution of the developed solution by comparing the two-stage method (unsupervised + supervised) with the supervised-only method. Due to severe class imbalance in SCADA data, the supervised model exhibits bias toward normal samples, leading to low fault-detection performance. In contrast, the two-stage method first applies the unsupervised OCSVM model to identify candidate anomaly samples, and then trains the CNN model only on these samples. As a result, the dominance of the normal samples is reduced, and the CNN model is trained to focus on abnormal samples of interest. It is noted that the Stage-1 OCSVM achieved 100% fault coverage on the testing data, and all the missed faults reported in Table 5 originate from the Stage-2 CNN classifier rather than the unsupervised screening stage.

Experimental results in Table 7 indicate that the supervised-only method achieved lower accuracy and suffered from higher missed normal/fault rates. The two-stage method significantly improved the fault detection performance, achieving lower missed fault rates and missed normal rate, and higher accuracy.

After training, the inference of the combined OCSVM-CNN is less than 100 ms per sample on a typical CPU, which is much shorter compared with the 10-min sampling interval of typical SCADA datasets. Even for SCADA datasets that have a 1-min sampling rate, the additional computational cost of 100 ms does not impact the real-time deployment of the framework. In other words, the two-stage framework provides a practical online condition monitoring in utility-scale wind farms. Overall, this study highlights that incorporating an unsupervised stage before a supervised stage allows handling class imbalance and improves the effectiveness of supervised learning in fault detection.

### 5.3. Transferability Across Datasets from Similar Turbines

**Figure 11:**
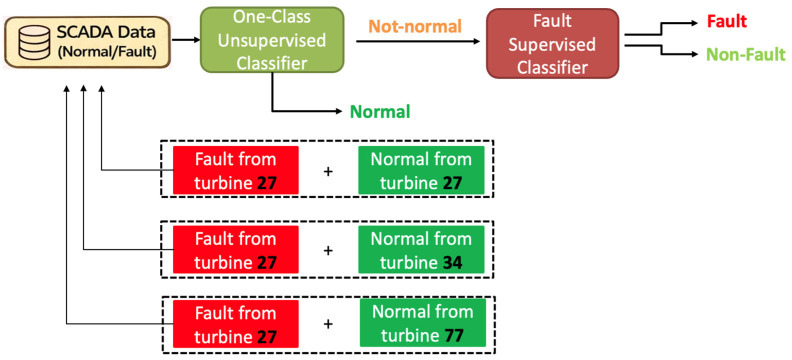
Illustration of cross-turbine transferability of normal data (faults from turbine #27 and normal data from the other turbines).

In this study, the transferability of the two-stage method across different turbines within the same wind farm (same make/model) was examined. Specifically, fault data from one turbine was considered with normal data from another turbine, and the above experiments were repeated. Since the turbines are of the same make/model and operate under similar conditions, their normal behavior is expected to produce similar normal SCADA data as mentioned in [29]. To evaluate the transferability of the proposed framework, fault samples from one turbine were combined with normal-operation samples from different turbines of the same make and model. For example, fault windows from turbine 27 were paired with normal windows from turbines 27, 34, and 77. This setup enables the investigation of whether fault characteristics learned from one turbine can be generalized to other turbines operating under similar conditions. Figure 11 provides an illustration of this transferability. The results of this study are shown in Table 8.

Although using normal data from other turbines leads to a slight reduction in accuracy compared to using data from the same turbine, the fault-detection capability remains high. In other words, the two-stage method remained effective under this cross-turbine setting, indicating the transferability of the fault detection framework when trained using normal data from similar make/model turbines. This finding has several important practical implications. First, if normal data from a specific turbine is unavailable, normal data from other turbines can be used without significantly affecting performance. Second, by using fault data from multiple turbines, it is possible to build a more generalizable fault detection classifier, reducing the need for turbine-specific training and lowering computational cost. Third, the unsupervised classifier trained solely on normal data can generate reliable abnormal alarm signals that serve as useful references even when fault labels are incomplete or unavailable.

Although the developed fault detection framework has demonstrated promising results, it is worth stating here its limitations. First, the anomaly selection threshold study and the transferability study were conducted using turbines of the same make and model. The optimal threshold and transferability may vary across different turbines and operating environments. In addition, while the fault detection framework effectively reduces the impact of class imbalance, fault types with limited samples would still remain a challenging task for supervised classifiers.

## 6. Conclusions

This paper has presented a two-stage method for detecting faults based on SCADA data. This method combines unsupervised anomaly detection or classification and supervised classification to address the problems of a lack of labeled data and severe class imbalance. In the first stage, the unsupervised classifier of OCSVM, together with the two feature-based and correlation-based anomaly scores, is used to identify abnormalities based on only normal SCADA data. In the second stage, the supervised classifier of CNN is applied to abnormal samples to identify fault samples among them. Experimental results show that the introduced two-stage detection method outperforms supervised-only methods, achieving higher accuracy and lower missed fault rates. Cross-turbine experiments further demonstrate that normal behavior learned from one turbine can be transferred to other turbines. Overall, the developed two-stage method provides improved wind turbine fault detection based on SCADA data.

## Figures and Tables

**Table 1:** Number of samples.

Turbine Number	# Training Samples (Normal/Fault)	# Validation Samples (Normal/Fault)	# Testing Samples (Normal/Fault)
27	36,633/134	5233/19	10,467/39
34	36,568/199	5224/28	10,449/57
77	36,669/98	5238/14	10,478/28

**Table 2:** Original features and their corresponding merged features.

Original Features	Merged Features
RMS voltage L1, L2, L3	Voltage_RMS
RMS current L1, L2, L3	Current_RMS
Generator stator winding temperature phase 1, 2, 3	Gen_Phase_Temp
HV transformer temperature L1, L2, L3	HVTrafo_Phase_Temp
Generator bearing 1, Generator bearing 2	Gen_Bear_Temp
Drive train vibration Z, Tower vibration X, Y	Tower_Train_Vibration
Gearbox oil inlet temperature, oil tank temperature	Gearbox_Temp

**Table 3:** Sensitivity Analysis of Anomaly-Selection Threshold.

Threshold	Fault Coverage
0.5%	5%
1%	16%
1.5%	26%
2%	58%
2.5%	94%
3%	94%
4%	94%
5%	94%

**Table 4:** Performance of unsupervised models and normal behavior models on fault coverage rate.

Unsupervised Model or NBM	T27 Fault Coverage Rate (%)	T34 Fault Coverage Rate (%)	T77 Fault Coverage Rate (%)
OCSVM	100	100	100
LSTM-AE	73.7	35.7	35.7
Autoencoder	84.2	21.4	28.6
Linear Regression	55.7	37.7	70.0
Unsupervised graph learning	10.5	0	42.9

**Table 5:** Performance of supervised models for fault detection.

Supervised Model	Missed Normal Samples (FP)			Missed Faults (FN)		
	T27	T34	T77	T27	T34	T77
CNN	1	4	5	0	1	0
XGboost	1	1	1	1	2	0
Random forest	0	0	0	0	2	2
LinearSVC	4	15	2	4	5	0
KNN	1	7	4	2	2	0
ConvLSTM + Transformer	2	2	6	0	3	0

**Table 6:** Comparison of fault coverage between feature-based and combined anomaly scores.

Turbine	Method	Fault Coverage Rate
27	Feature-based anomaly score	100%
	Combined anomaly scores	100%
34	Feature-based anomaly score	98%
	Combined anomaly scores	100%
77	Feature-based anomaly score	91%
	Combined anomaly scores	100%

**Table 7:** Fault detection performance using the supervised method vs. the two-stage method.

Turbine	Method	Missed Normal/ Total Normal	Missed Faults/ Total Faults	Overall Accuracy
27	CNN	70/10,467	4/39	99.3%
	CNN + OCSVM	9/10,467	2/39	99.9%
34	CNN	193/10,449	7/57	98.1%
	CNN + OCSVM	13/10,449	0/57	99.9%
77	CNN	64/10,478	2/28	99.4%
	CNN + OCSVM	7/10,478	2/28	99.9%

**Table 8:** Performance comparison using normal data from other turbines.

Turbine	Normal Data Source	Missed Normal/ Total Normal	Missed Faults/ Total Faults	Overall Accuracy
27	27	9/10,467	2/39	99.9%
	34	16/10,467	5/39	99.8%
	77	16/10,467	3/39	99.8%
34	27	8/10,449	6/57	99.9%
	34	13/10,449	0/57	99.9%
	77	12/10,449	4/57	99.8%
77	27	32/10,478	5/28	99.6%
	34	15/10,478	6/28	99.8%
	77	7/10,478	2/28	99.9%

## Data Availability

The dataset used in this study is publicly available through the Zenodo repository stated in Reference [21].

## References

[1] Ember. https://ember-climate.org/topics/wind/.

[2] Dai J., Rotea M., Kehtarnavaz N. (2025). Obtaining Rotational Stiffness of Wind Turbine Foundation from Acceleration and Wind Speed SCADA Data. Sensors.

[3] Singh U., Rizwan M. (2023). Analysis of Wind Turbine Dataset and Machine Learning Based Forecasting in SCADA-System. J. Ambient. Intell. Humaniz. Comput..

[4] Singh U., Rizwan M. (2022). SCADA System Dataset Exploration and Machine Learning Based Forecast for Wind Turbines. Results Eng..

[5] Schlechtingen M., Santos I.F., Achiche S. (2013). Wind Turbine Condition Monitoring Based on SCADA Data Using Normal Behavior Models. Part 1: System Description. Appl. Soft Comput..

[6] Meyer A. (2021). Multi-Target Normal Behaviour Models for Wind Farm Condition Monitoring. Appl. Energy.

[7] Vidal Y., Pozo F., Tutivén C. (2018). Wind Turbine Multi-Fault Detection and Classification Based on SCADA Data. Energies.

[8] Li G., Wang C., Zhang D., Yang G. (2021). An Improved Feature Selection Method Based on Random Forest Algorithm for Wind Turbine Condition Monitoring. Sensors.

[9] Trizoglou P., Liu X., Lin Z. (2021). Fault Detection by an Ensemble Framework of Extreme Gradient Boosting (XGBoost) in the Operation of Offshore Wind Turbines. Renew. Energy.

[10] Xiang L., Wang P., Yang X., Hu A., Su H. (2021). Fault Detection of Wind Turbine Based on SCADA Data Analysis Using CNN and LSTM with Attention Mechanism. Measurement.

[11] Rama V.S.B., Hur S.-H., Yang J.-M. (2024). Short-Term Fault Prediction of Wind Turbines Based on Integrated RNN-LSTM. IEEE Access.

[12] Delgado I., Fahim M. (2021). Wind Turbine Data Analysis and LSTM-Based Prediction in SCADA System. Energies.

[13] Liu X., Du J., Ye Z.-S. (2022). A Condition Monitoring and Fault Isolation System for Wind Turbine Based on SCADA Data. IEEE Trans. Ind. Inform..

[14] Jin X., Pan H., Ying C., Kong Z., Xu Z., Zhang B. (2022). Condition Monitoring of Wind Turbine Generator Based on Transfer Learning and One-Class Classifier. IEEE Sens. J..

[15] Tutivén C., Vidal Y., Insuasty A., Campoverde-Vilela L., Achicanoy W. (2022). Early Fault Diagnosis Strategy for WT Main Bearings Based on SCADA Data and One-Class SVM. Energies.

[16] McKinnon C., Carroll J., McDonald A., Koukoura S., Infield D., Soraghan C. (2020). Comparison of New Anomaly Detection Technique for Wind Turbine Condition Monitoring Using Gearbox SCADA Data. Energies.

[17] Ferreira V.Z., Brito E.M., Da Cunha G.M.F., Perez F.A.R., Dos Reis E.D.W., Pereira E.R., Louzada F. (2026). Unsupervised Autoencoder-Based Anomaly Detection Under Limited Failure Data for Oil Industry. IEEE Open J. Instrum. Meas..

[18] Chen H., Liu H., Chu X., Liu Q., Xue D. (2021). Anomaly Detection and Critical SCADA Parameters Identification for Wind Turbines Based on LSTM-AE Neural Network. Renew. Energy.

[19] Seifert J.K., Kraft M., Kühn M., Lukassen L.J. (2021). Correlations of Power Output Fluctuations in an Offshore Wind Farm Using High-Resolution SCADA Data. Wind. Energy Sci..

[20] Maldonado-Correa J., Valdiviezo-Condolo M., Artigao E., Martín-Martínez S., Gómez-Lázaro E. (2024). Classification of Highly Imbalanced Supervisory Control and Data Acquisition Data for Fault Detection of Wind Turbine Generators. Energies.

[21] NIAID Data Discovery Portal Wind Turbine SCADA Data for Early Fault Detection. https://data.niaid.nih.gov/resources?id=zenodo_10958774.

[22] Zhang F., Wen Z., Liu D., Jiao J., Wan H., Zeng B. (2020). Calculation and Analysis of Wind Turbine Health Monitoring Indicators Based on the Relationships with SCADA Data. Appl. Sci..

[23] Dao P.B. (2022). Condition Monitoring and Fault Diagnosis of Wind Turbines Based on Structural Break Detection in SCADA Data. Renew. Energy.

[24] Zhu Y., Xie B., Wang A., Qian Z. (2025). Wind Turbine Fault Detection and Identification via Self-Attention-Based Dynamic Graph Representation Learning and Variable-Level Normalizing Flow. Reliab. Eng. Syst. Saf..

[25] Fazli A., Poshtan J. (2024). Wind Turbine Fault Detection and Isolation Robust against Data Imbalance Using KNN. Energy Sci. Eng..

[26] Guo J., Song X., Tang S., Zhang Y., Wu J., Li Y., Jia Y., Cai C., Li Q. (2024). Fault Diagnosis of Wind Turbine Blade Icing Based on Feature Engineering and the PSO-ConvLSTM-Transformer. Ocean. Eng..

[27] Preciado-Grijalva A., Iza-Teran V.R. (2021). Anomaly Detection of Wind Turbine Time Series Using Variational Recurrent Autoencoders. arXiv.

[28] Vásquez-Rodríguez G., Maldonado-Correa J. (2024). Anomaly-Based Fault Detection in Wind Turbines Using Unsupervised Learning: A Comparative Study. IOP Conf. Ser. Earth Environ. Sci..

[29] Ulmer M., Jarlskog E., Pizza G., Huber L.G. (2020). Cross-Turbine Training of Convolutional Neural Networks for SCADA-Based Fault Detection in Wind Turbines. Annu. Conf. PHM Soc..

